# Structure based virtual screening and molecular simulation study of FDA-approved drugs to inhibit human HDAC6 and VISTA as dual cancer immunotherapy

**DOI:** 10.1038/s41598-023-41325-9

**Published:** 2023-09-02

**Authors:** Muhammad Shahab, Haitham Al-Madhagi, Guojun Zheng, Amir Zeb, Abdullah Fayez Alasmari, Metab Alharbi, Fawaz Alasmari, Muhammad Qayash Khan, Momin Khan, Abdul Wadood

**Affiliations:** 1 State Key Laboratories of Chemical Resources Engineering, Beijing University of Chemical Technology, Beijing, 100029 China; 2 Biochemical Technology Program, Dhamar University, Dhamar, Yemen; 3 Department of Natural and Basic Science, Faculty of Science and Engineering, University of Turbat, Turbat, 92600 Pakistan; 4 Department of Pharmacology and Toxicology, College of Pharmacy, King Saud University, 11451 Riyadh, Saudi Arabia; 5 Department of Zoology, Abdul Wali Khan University Mardan, Mardan, 23200 Pakistan; 6 Department of Chemistry, Abdul Wali Khan University Mardan, Mardan, 23200 Pakistan; 7 Department of Biochemistry, Abdul Wali Khan University Mardan, Mardan, 23200 Pakistan

**Keywords:** Computational biology and bioinformatics, Drug discovery

## Abstract

Cancer immunotherapy has significantly contributed to the treatment of various types of cancers mainly by targeting immune checkpoint inhibitors (ICI). Among them, V-domain immunoglobulin suppressor of T cell activation (VISTA) has been explored as a promising therapeutic target. Besides, histone deacetylase 6 (HDAC6) has been demonstrated to be efficacious target for several cancers. The current theoretical work was performed to explore the virtual repurposing of the FDA-approved drugs as inhibitors against these two (VISTA and HDAC6) cancers therapeutic targets. The crystal structure of the two proteins were downloaded from PDB and subjected to virtual screening by DrugRep webserver while using FDA-approved drugs library as ligands database. Our study revealed that Oxymorphone and Bexarotene are the top-ranked inhibitors of VISTA and HDAC6, respectively. The docking score of Bexarotene was predicted as − 10 kcal/mol while the docking score of Oxymorphone was predicted as − 6.2 kcal/mol. Furthermore, a total of 100 ns MD simulation revealed that the two drugs Oxymorphone and Bexarotene formed stable complexes with VISTA and HDAC6 drug targets. As compared to the standard drug the two drugs Oxymorphone and Bexarotene revealed great stability during the whole 100 ns MD simulation. The binding free energy calculation further supported the Root Mean Square Deviation (RMSD) result which stated that as compared to the ref/HDAC6 (− 18.0253 ± 2.6218) the binding free energy score of the Bexarotene/HDAC6 was good (− 51.9698 ± 3.1572 kcal/mol). The binding free energy score of Oxymorphone/VISTA and Ref/VISTA were calculated as − 36.8323 ± 3.4565, and − 21.5611 ± 4.8581 respectively. In conclusion, the two drugs deserve further consideration as cancer treatment option.

## Introduction

Tumorigenesis is a multi-step successive process driven by DNA sequence mutations in the housekeeping genes leading to the transformation of cell biology succeeded by cell behavior and malignancy^1^. In addition to DNA sequence mutations, heritable epigenetic changes also account for the cancer-pattern establishment. One of the epigenetic alterations implicated in almost every aspect of cancer is histone acetylation which is catalyzed by histone acyltransferase histone deacetylase (HDAC). HDACs family constitutes many isoforms differing in location, associated genes regulation, and activity^2^. Due to the connection between aberrant HDAC enzyme expression and various cancers, clinical trials are assessing HDAC-specific inhibitors for corresponding cancer treatments^3^. For instance, ACY-1215 (ricolinostat), a specific HDAC6 inhibitor, is undergoing a phase 1b trial for relapsed/refractory multiple myeloma, while ACY-241, a second-generation HDAC6 inhibitor with improved properties, is being investigated for multiple myeloma treatment. These inhibitors, along with others like A452, are part of the effort to develop targeted therapies based on the potential of HDAC inhibition in cancer treatment. Some HDAC inhibitors have already gained FDA approval, underscoring their promise in this field^4–7^. Among them, HDAC6 is the predominant isozyme present in the cytoplasm whose primary role is to regulate tubulin^8^. HDAC6 is distinct for having two catalytic domains, CD1 and CD2, making it a valuable target for treating cancer and Alzheimer's disease^9,10^. Additionally, HDAC6 enhances key cancer immunotherapy targets, including PD-1 and its ligand PD-L1, crucial for immune checkpoint regulation^11^. This approach effectively addresses two goals: halting cancer progression and reversing immunosuppression, leading to enhanced leukocyte infiltration into tumor tissues^12^.

**Figure 1:**
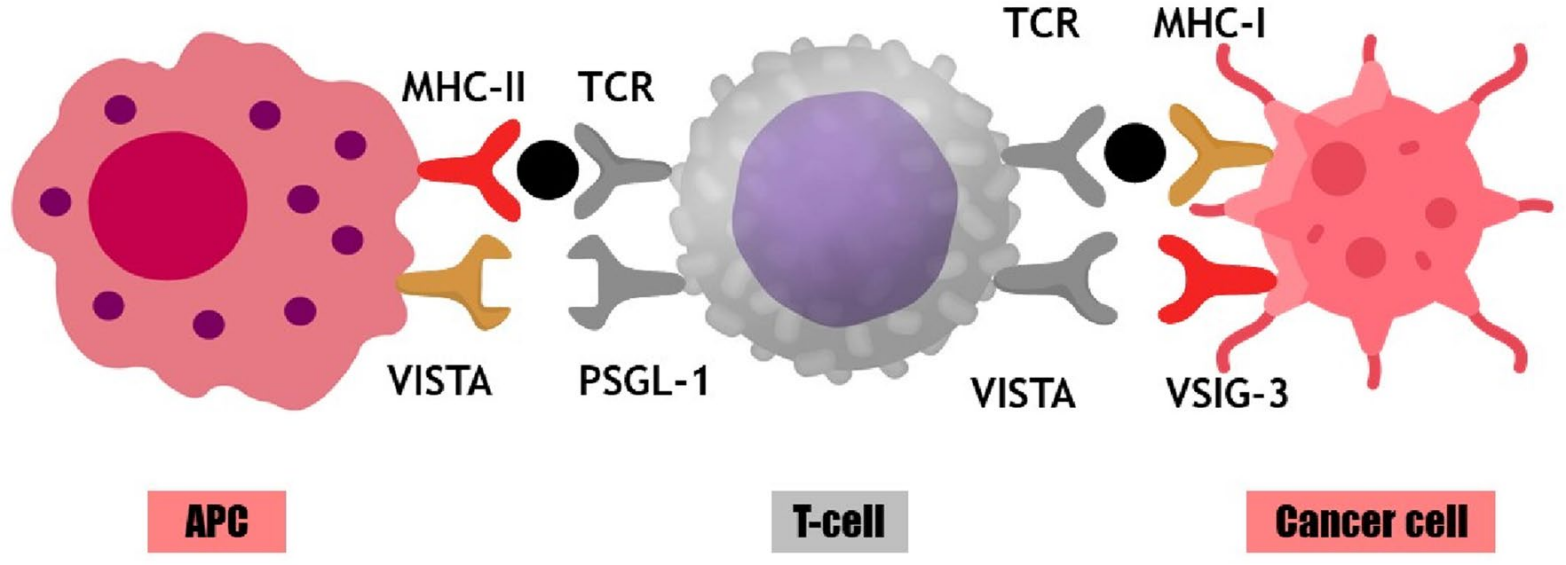
Interaction among antigen-presenting cell, T-cell and cancer cells by immune membrane receptors. *MHC* major histocompatibility complex, *PSGL-1* P-selectin glycoprotein ligand-1, *VSIG-3* V‐Set and immunoglobulin domain containing receptor 3.

VISTA (V-domain immunoglobulin suppressor of T cell activation) is a transmembrane receptor that resembles an immunoglobulin 3D structure^13^. Similarly, elevated VISTA levels are observed in late-stage oral squamous cell carcinomas, gastrointestinal and prostate cancers, contrasting with lower levels in their early stages^14^. It is highly expressed in hematopoietic cells in their two lineages: myeloid and lymphoid. Myeloid cells-expressing VISTA includes neutrophils and to a lesser extent monocytes and macrophages whereas naïve CD4^+^ and Foxp3^+^ regulatory T cells constitute the lymphoid lineage^15^. VISTA has gained wide attention as a promising therapeutic target because it poses co-stimulatory action in immune checkpoint inhibition (ICI) pathway. Indeed, majority of attention is paid not only to its ICI implication but also to its folded structure^16^. As a transmembrane receptor, it is composed of 3 domains: N-terminal V-domain which is very similar to PD-L1, a transmembrane domain, and a long cytoplasmic tail which resembles CD28 and CTLA-4^17^. The role of VISTA in immune response regulation is complex and controversial. VISTA not only acts as a ligand expressed on antigen-presenting cells (APC), but also serves as a receptor on T cells (Fig. 1). To date, most studies have described the suppressive effect of VISTA on the immune system and the ability of VISTA-deficiency or anti-VISTA treatment to upregulate immune responses^18^. JNJ-61610588 is a human monoclonal antibody targeting VISTA, in clinical trials for advanced cancer. It blocks VISTA signalling, enhancing T cell responses against tumors and inhibiting tumor growth. Meanwhile, a study of CA-170 (VISTA inhibitor) is still currently being conducted in advanced solid tumors or lymphomas^19^. The aim of this study aims to fill is the exploration of dual targeting, where both HDAC6 and VISTA are simultaneously inhibited by FDA-approved drugs. While each of these targets has been individually studied as potential therapeutic avenues, combining their inhibition could offer a synergistic effect, potentially leading to enhanced treatment outcomes. However, the exact interplay between these two targets and the potential for enhanced efficacy remain largely unexplored. By leveraging computational techniques such as, molecular docking, and MD simulation has become an increasingly important tool for drug discovery, this study offers a rapid and cost-effective way to screen a vast library of FDA-approved drugs and identify potential candidates with dual inhibitory activity against HDAC6 and VISTA. This process aids in the identification of potential candidates displaying dual inhibitory capabilities against HDAC6 and VISTA. Enhancing the scoring function, a fundamental aspect of docking, holds significance, as molecular docking methods exhibit the ability to comb through vast databases for hit identification and novel small molecule design^20,21^. It could offer a personalized medicine approach by tailoring existing drugs for specific patients, taking into account their unique molecular profiles and therapeutic needs.

## Material and methods

**Figure 2:**
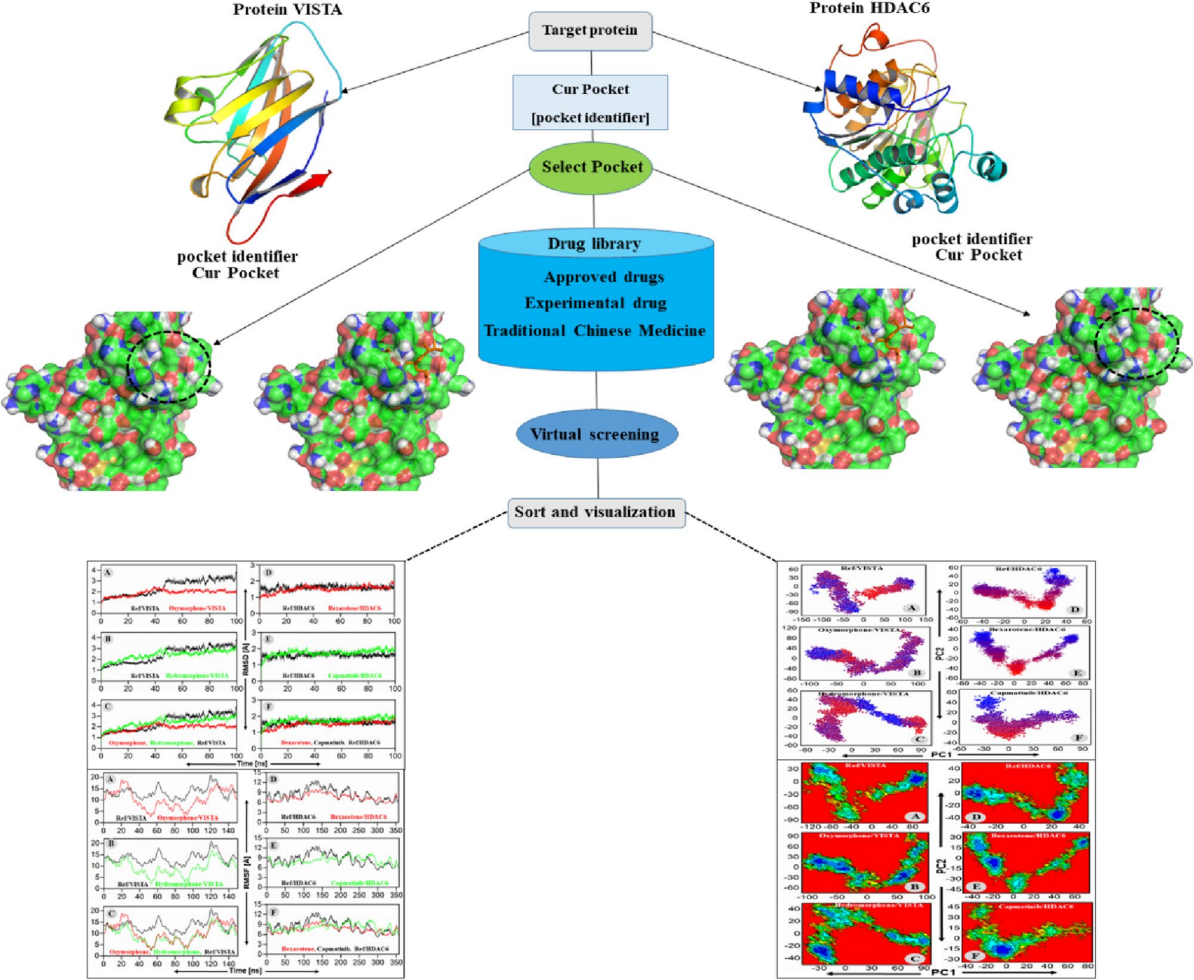
Step-wise workflow of the present study.

Overall workflow of the present study is displayed in Fig. 2.

### Receptor preparation for virtual screening

In this study, two proteins i.e. HDAC6 and VISTA were targeted. The experimentally determined 3-D structures of both the target proteins (HDAC6 and VISTA) were retrieved from Protein Data Bank (https://www.rcsb.pdb.org/). The PDB IDs for HDAC6 and VISTA structures are 6OIL and 5EF8, respectively^14,15^. The structures of both the target proteins were prepared by adding the polar hydrogen atoms and other missing atoms, removing co-crystalized water and heteroatoms and assigning partial charges and their energies were minimized by employing the conjugate gradient and steepest descent algorithms of UCSF Chimera v1.16^22^. Since, the structure of HDAC6 is comprised of two chains, only chain A was selected during the docking operations.

### Ligands library preparation for virtual screening

For ligands library preparation, the FDA-approved drugs library (2315 drugs) was selected^23^. All the FDA-approved drug molecules were downloaded from Drug Bank (https://go.drugbank.com/) in structured data file sdf format. The structures of FDA-approved drug molecules were energy minimized and prepared as ligands library for virtual screening. This library was selected as we purposed to perform drug repurposing of FDA-approved drugs.

### Virtual screening

Herein, the virtual screening was executed by the recently developed server DrugRep^24,25^. DrugRep is a programmed and parameter-free online virtual screening server which implants AutoDock Vina algorithm for multiple ligands docking and rank the best fitted conformers of molecules by computing the docking score, binding conformation and binding affinity between the receptor and ligands structures. This server also provides pharmacodynamics services including the prediction of absorption, distribution, metabolism and excretion (ADME) properties of the top ligands^26^. During the virtual screening by DrugRep, the previously well-prepared receptors’ structures were subjected to structure-based virtual screening of the previously prepared FDA-approved drug library which contains 2315 different drug molecules (http://cao.labshare.cn:10180/DrugRep/php/index.php)^24^. During the docking protocol, docking grids were generated for both the receptors including HDAC6 and VISTA. The docking grid of HDAC6 was extended enough to include almost all the binding site residues of the pocket 4 including residues Phe643, Phe642, Ser531, Gly582, Tyr745, Hsd574, Hsd614, Leu712, and Phe583. The docking grid of VISTA was architecture in such a way that it spanned the binding site residues of pocket 8 including residues Ser151 Gly130 Hsd129 Arg45 Ser8 Ala131 Val3 and Thr5.

### Receptor–ligand complex analyses

To elucidate the pattern of interactions between the receptors and top-ranked drugs, Discovery Studio Visualizer 2022 was used^27^. Both receptors in complex with their respective ligands (drug molecules) were analyzed for their interactions, binding conformation, and binding affinity (docking score). The complexes were also analyzed for their pharmacokinetics characteristics including molecular weight, number of hydrogen bond donors and acceptors and LogP values (Lipinski’s rule of five)^28^. The best fitted ligands in terms of binding affinity were selected on their best docking score, pharmacokinetics and pharmacodynamics properties.

### Molecular dynamics simulation

The molecular dynamic simulation (MDS) was performed to investigate the dynamic behavior of proteins upon inhibitor binding at the atomistic level. MDS was employed to assess the stability of the compounds that had previously been recovered at the active sites of (HDAC6 and VISTA) complex. Six compounds were chosen based on their favorable interactions and docking scores. Molecular Dynamics (MD) simulations were conducted for these compounds, with three selected for each HDAC6 and VISTA protein, representing the best-fitting ligands. In case of HDAC6, the MD simulations were performed on the docked conformations of the Bexarotene, Capmatinib, and the reference compound inside the active site of the HDAC6. In case of VISTA, the MD simulations were performed on the docked conformations of the Oxymorphone, Hydromorphone and the reference compound in the active site of the VISTA protein. All simulations were performed using the ff19SB force field and the Amber22 package^29^. During MDS, all the complexes were constructed and solved using the preparation program Tleap. Herein, a solvated octahedral box was utilized. Upon solvating each systems using the TIP3P water model in an octahedral box, the complexes were neutralized by introducing counterions (Na^+^ or Cl^-^). In order to relaxing all systems, the energy of each neutralized system was minimized as much as feasible in two steps. The parameters for energy minimization included a maximum of 5000 steps using the steepest descent algorithm, followed by conjugate gradient optimization until a convergence criterion of 0.1 kcal/(mol Å) was met. The complexes after minimization were heated up to 300 K for 50 ps. Then, using a two-step process at constant pressure (1 atm) and temperature (298 K), each system was brought to equilibrium. First, a weak constraint was utilized to equilibrate the density for 50 ps. Next, we equilibrated the system for 1 ns without any restrictions. The production phase was then run for 100 ns. The pressure of each system was monitored using the Berendsen barostat, and the temperature was kept stable using the Langevin thermostat^30,31^. We applied the AMBER22 Particle Mesh Ewald (PME) algorithm to calculate long-range electrostatic interactions. Van der Waals interactions and long-range electrostatic interactions both had a cut-off distance of 10 Å. The AMBER22 SHAKE algorithm was used to refine the covalent bonds^32^. All the simulations were performed using the GPU version (PMEMD.cuda) of AMBER22. The MD trajectories were examined using the AMBER22 CPPRTAJ module, PyMol v1.7, and Origin Pro Lab v2018. The interface analysis and graphical representation were completed.

### Evaluation of the binding free energy

Trajectories were made with the help of MDS run while executing the MMPBSA.py script, which were then employed in the estimation of binding free energy (BFE)^33^. Several studies have used this method to figure out the free energies of binding for P–P (protein–protein), protein–ligand, and nucleic acid–protein complexes^26^. The total free energy of binding, de-noted by the symbol Gbind, was determined with the assistance of the following equation:

$$ \Delta {\text{Gbind }} = \, \Delta {\text{Gcomplex}} - \, \left[ {\Delta {\text{Greceptor }} + \, \Delta {\text{Gligand}}} \right] $$

In order to gain a better understanding of how each of the energy terms, such as polar (Gpol), van der Waal forces (GvdW), electrostatic energy (Gele), no-polar interactions (Gnpol), Gbond showed the angle of bond and their dihedral energy, TS represents the absolute temperature (T), and entropy (S), contribute to the total energy, the following equation was utilised (G).

$$ {\text{G }} = {\text{ Gele }} + {\text{ Gbond }} + {\text{ GvdW }} + {\text{ Gnpol }} + {\text{ Gpol }}{-}{\text{ TS}} $$

The binding free energies of the obtained complexes were calculated using the molecular mechanics generalized born surface area (MMGBSA) approach. The lower the value of the MMGBSA, which is a BFE index, the stronger the bond. The python script MMGBSA.py was used to determine the binding free energy of the obtained and standard complexes in Amber22^34^. For complexes, the stability of the system was demonstrated by a decline in potential energy over 100 ns. After simulating for 100 ns, we look at the different shapes that emerge. In or-der to determine the BFE between receptor and the returned hits and the reference medicine, the MMGBSA.py script was utilized^35^. The binding free energy (BFE) of the ligand–protein complex was evaluated using the “MMGBSA” methodology. To verify its accuracy, the results were compared and validated against the reference values for both VISTA and HDAC6. This analysis was conducted using the AMBER22 software along with the AMBER Tools MMGBSA scripts^36^.

## Results

### Virtual screening

Virtual screening of FDA-approved drug library which contains 2315 different drug molecules (http://cao.labshare.cn:10180/DrugRep/php/index.php) was carried out by DrugRep server. According to our docking results, Bexarotene strongly bonded HDAC6 and obtained lowest docking score (− 10 kcal/mol) (Table 1). The docking score of the top-10 drugs were − 10 to − 8.9 kcal/mol in the active site of HDAC6, which reflect their high binding energy towards the HDAC6. With respect to Lipinski's rule of five (RO5), only the best model (Bexarotene) has a single violation regarding the LogP greater than the optimal range (< 5), which should not affect the pharmacokinetics characteristics of the drug since it is already FDA-approved. The remaining drugs could strictly follow the RO5. Overall, as compared to the reference compound of HDAC6, all the top-10 drug candidates exhibited high affinity towards the HDAC6. According to our docking results, Bexarotene strongly bonded to HDAC6 and obtained lowest docking score (− 10 kcal/mol) (Table 1). The docking score of the top-10 drugs were − 10 to − 8.9 kcal/mol in the active site of HDAC6, which reflect their high binding energy towards the HDAC6. With respect to Lipinski's rule of five (RO5), only the best model (Bexarotene) has a single violation regarding the LogP greater than the optimal range (< 5), which should not affect the pharmacokinetics characteristics of the drug since it is already FDA-approved. The remaining drugs could strictly follow the RO5. Overall, as compared to the reference compound of HDAC6, all the top-10 drug candidates exhibited high affinity towards the HDAC6.

**Table 1:** Binding affinities as well as Lipinski's rule of five of top-10 HDAC6 inhibitors.

Drug name	Score	MW	HBD	HBA	RB	Rings	LogP
Bexarotene	− 10	348.4779	1	2	4	3	7.6
Capmatinib	− 9.5	412.428	1	5	5	5	2.9
Belinostat	− 9.4	318.35	3	4	7	2	1.6
Cyproheptadine	− 9.3	287.3981	0	0	0	4	4.2
Oxyphenisatin acetate	− 9.2	401.418	1	3	6	4	3.4
Acrivastine	− 9.2	348.4382	1	3	5	3	3.1
Ketotifen	− 9.1	309.425	0	1	0	4	3.2
Phenolphthalein	− 9.1	318.3228	2	3	4	4	3.8
Panobinostat	− 8.9	349.434	3	2	9	3	2.9
Bisoxatin	− 8.9	333.343	3	3	4	4	3.3
Reference inhibitor	− 8.1	302.37	4	2	6	1	2.7

*MW* molecular weight, *HBD* hydrogen bond donor, *HBA* hydrogen bond acceptor, *RB* rotatable bonds.

Furthermore, the binding energy of the top-10 drugs towards the VISTA receptor was significantly lower than the top-10 drugs of HDAC6 against the HDAC6 (Table 2). The energy range of the top-10 drugs was found − 6.2 to − 5.7 kcal/mol. It should be noted that, after docking validation, Oxymorphone binding affinity was elevated to be − 7.2 kcal/mol making it the best potential VISTA inhibitor. RO5 of all top-10 drugs are within the desired range except for Zanamivir which has 11 rotatable bonds (desired < 10) (Table 2).

**Table 2:** Binding affinities as well as Lipinski's rule of five of top-10 VISTA inhibitors.

Drug Name	Score	MW	HBD	HBA	RB	Rings	LogP
Oxymorphone	− 6.2	301.3371	2	3	2	5	0.7
Hydromorphone	− 6	285.3377	1	2	1	5	1.8
Morphine	− 5.9	285.3377	2	2	2	5	1.5
Spectinomycin	− 5.9	332.3496	5	4	5	3	− 3
Zanamivir	− 5.8	332.3098	7	6	11	1	− 3.1
Naloxone	− 5.8	327.3743	2	3	4	5	1.4
Saxagliptin	− 5.7	315.41	2	3	4	5	0.7
Pirenzepine	− 5.7	351.4023	1	3	3	4	0.9
Dicoumarol	− 5.7	336.295	2	4	4	4	1.6
Diamorphine	− 5.7	369.411	0	2	4	5	2.2
Reference inhibitor	− 5.8	374.35	7	10	10	1	− 6.7

### Receptor–ligand interaction analyses

Our study shortlisted the top-10 drug molecules that have obtained lowest docking score with HDAC6 (Table 1). The interaction analyses of the top-3 drugs suggested that drug A formed two hydrogen bonds with Asp612 and His614, drug C formed one hydrogen bond with Gly154, while the reference compound E of HDAC6 has established three hydrogen bonds with Gly154, Phe210, and His183 of the HDAC6 (Fig. 2). Apart from polar interactions (hydrogen bonds), all the three drugs also established an extensive nonpolar (hydrophobic) interactions network with several residues of the HDAC6 binding pocket (Fig. 2).

**Figure 3:**
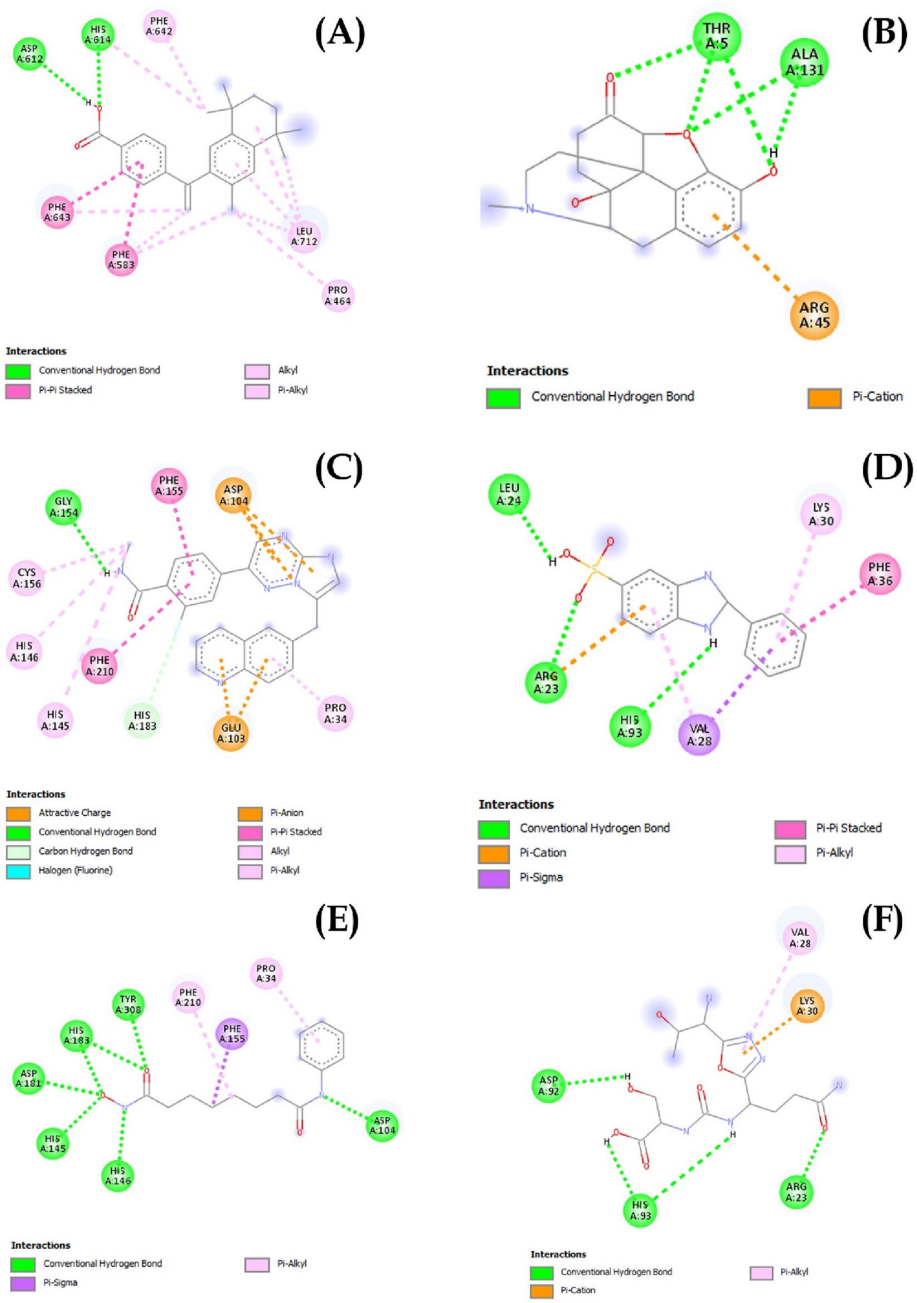
Receptor (HDAC6 and VISTA)–ligand interactions pattern visualized by Discovery studio. (**A**) and (**C**) refer to top 2 HDAC6 inhibitors whereas (**B**) and (**D**) refer to top 2 VISTA inhibitors. (**E**) and (**F**) refer to reference inhibitors of HDAC6 and VISTA, respectively.

In parallel, the interaction pattern analyses of the top-2 drugs against the VISTA (Table 2) suggested that drug B formed four hydrogen bonds with Thr5 and Ala131 of VISTA, while the drug D formed three hydrogen bonds with Leu24, Arg23 and His93 of the VISTA receptor (Fig. 3). In contrast, the reference compound “F” of VISTA receptor couldn’t establish a polar interaction with the binding pocket residues of VISTA during the docking operation (Fig. 3). Similarly, several nonpolar (hydrophobic) interactions between the binding site residues of the VISTA receptor and each selected drug were established (Fig. 3). The best drug candidate for HDAC6 (Bexarotene) was investigated for its interaction with the receptor in order to identify the catalytic residues implicated in bonding. His 573 and His 614 formed 2 H-bonds with the carboxylate oxygen atoms while Asp 612 and 705 stabilizes the interaction through forming H-bonds with the catalytic His residues. Furthermore, 3 Phe, 2 Gly along with Leu 712, Tyr 745, Ser 531 and His 574 contributed to the bonding by hydrophobic attraction as illustrated in Fig. 2. Oxymorphone, on the other hand, was H-bonded to active site residues Thr 5 and Ala 131 while the rest of interactions was contributed by Val 3, Ser 8, His 129 and Gly 130 hydrophobic (Fig. 2).

### Molecular dynamics simulation analyses

#### Root means square deviation (RMSD) analyses

**Figure 4:**
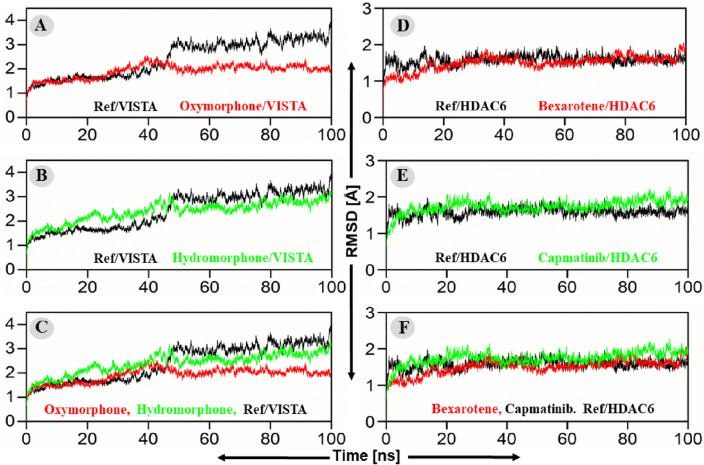
(**A**–**C**) RMSD Plot of the repurposed drugs/VISTA and ref-drug (CA-170)/VISTA complexes. The x-axis and y-axis showed the time in nanoseconds (100 ns) and RMSD in Angstroms respectively. (**A**) RMSD of Oxymorphone–VISTA complex (red) and CA-170/VISTA complex (black), (**B**) Hydromorphone–VISTA complex (Green) and CA-170/VISTA complex (Black) RMSD, (**C**) Combine RMSD compassion of VISTA. While (**D**–**F**) plot of the hits/HDAC6 and ref-drug (Trichostatin A)/HDAC6 complexes’ root mean square deviations. (**D**) RMSD of Bexarotene–HDAC6 complex (red) and CA-Trichostatin A/HDAC6 (black), (**E**) RMSD of Capmatinib–HDAC6 complex (Green) and CA-Trichostatin A/HDAC6 (black), (**F**) Combine RMSD compassion of HDAC6.

The MDS results suggested two drugs named Oxymorphone and Bexarotene that could be the dual inhibitors of HDAC6 and VISTA. The result of MD simulation suggested that as compared to the reference compounds, the root means square deviation (RMSD) of VISTA in complex with Oxymorphone was converged right after 5 ns of the simulation and remained stable throughout the simulation period with an average RMSD value of 1.9 Å (Fig. 4A). The VISTA in complex with Hydromorphone also converged right after 5 ns of simulation and could remain stable during the entire simulation period with an average value of 2.5 Å with a slight deviation at the end of the simulation (Fig. 3, left panel). The superimposition of all the three simulated systems (reference, Oxymorphone and Hydromorphone in complex with VISTA) suggested that Oxymorphone behaved more stable which is followed by Hydromorphone as compared to the reference compound of the VISTA (Fig. 4C, left panel).

Afterwards, the result of MD simulation suggested that as compared to the reference compounds, the root mean square deviation (RMSD) of HDAC6 in complex with Bexarotene was converged right after the 2 ns of simulation and then retained stable throughout the simulation period with an average value of 1.15 Å (Fig. 4D). In parallel, HDAC6 in complex with Capmatinib also converged right after 2 ns of simulation and could remained stable during the entire simulation period with an average value of 2.5 Å RMSD as shown in Fig. 4. The superimposition of all the three simulated systems (reference, Bexarotene and Capmatinib in complex with HDAC6) suggested that both the drugs obtained same stability as compared to the reference compound of the HDAC6 (Fig. 4, right panel).

#### Root means square fluctuation analyses

**Figure 5:**
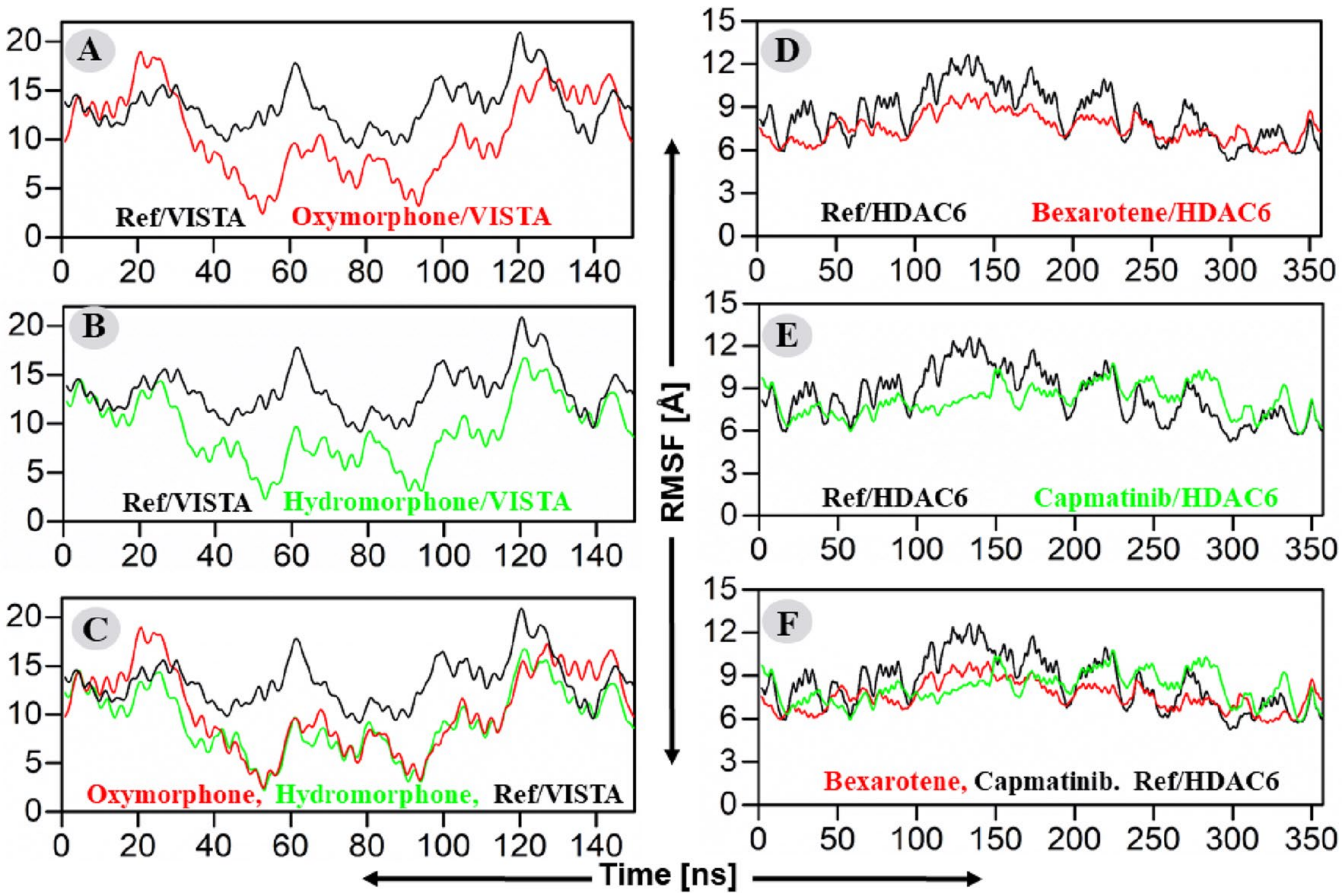
(**A**–**C**) RMSF plots of the repurposed drugs/VISTA and ref-drug (CA-170). The x-axis and y-axis showed the time in nanoseconds (100 ns) and RMSF in Angstroms respectively. (**A**) RMSF of Oxymorphone–VISTA complex (red) and CA-170/VISTA complex (black), (**B**) Hydromorphone–VISTA complex (Green) and CA-170/VISTA complex (Black) RMSF, (**C**) Combine RMSF compassion of VISTA. While (**D**–**F**) plot of the hits/HDAC6 and ref-drug (Trichostatin A)/HDAC6 complexes’ root mean square deviations. (**D**) RMSF of Bexarotene–HDAC6 complex (red) and CA-Trichostatin A/HDAC6 (black), (**E**) RMSD of Capmatinib–HDAC6 complex (Green) and CA-Trichostatin A/HDAC6 (black), (**F**) Combine RMSD compassion of HDAC6.

Root means square fluctuation (RMSF) is an important parameter that describes the flexibility of each residues of the receptor. The RMSF analyses reflect the stability of the receptor in general and the fluctuation of the binding pocket residues in particular. The RMSF analyses of our result suggested that the reference compound of VISTA receptor showed high fluctuation (average value ~ 15 Å) as compared to the fluctuation observed for the complex of VISTA and Oxymorphone (average value ~ 8.5 Å) (Fig. 5A, left panel). In parallel, the Hydromorphone also exhibited lowest RMSF (average value ~ 8 Å) with VISTA (Fig. 5B, left panel). The comparative analyses suggested that both Oxymorphone and Hydromorphone remained more stable (average RMSF value ~ 8.2 Å) as compared to the reference compound of the VISTA protein (Fig. 5C). The RMSF analyses of HDAC6 and the candidate inhibitors suggested that the reference compound showed high fluctuation (average value ~ 8 Å) as compared to the fluctuation observed for the complex of HDAC6 and Bexarotene (average value ~ 7 Å) (Fig. 5D, right panel). In parallel, the Capmatinib also exhibited lowest RMSF (average value ~ 7 Å) with VISTA (Fig. 5E, right panel). The comparative analyses suggested that both Bexarotene and Capmatinib remained more stable (average RMSF value ~ 7 Å) as compared to the reference compound of the HDAC6 protein (Fig. 5F).

#### Hydrogen bonds (polar interactions) analyses

**Figure 6:**
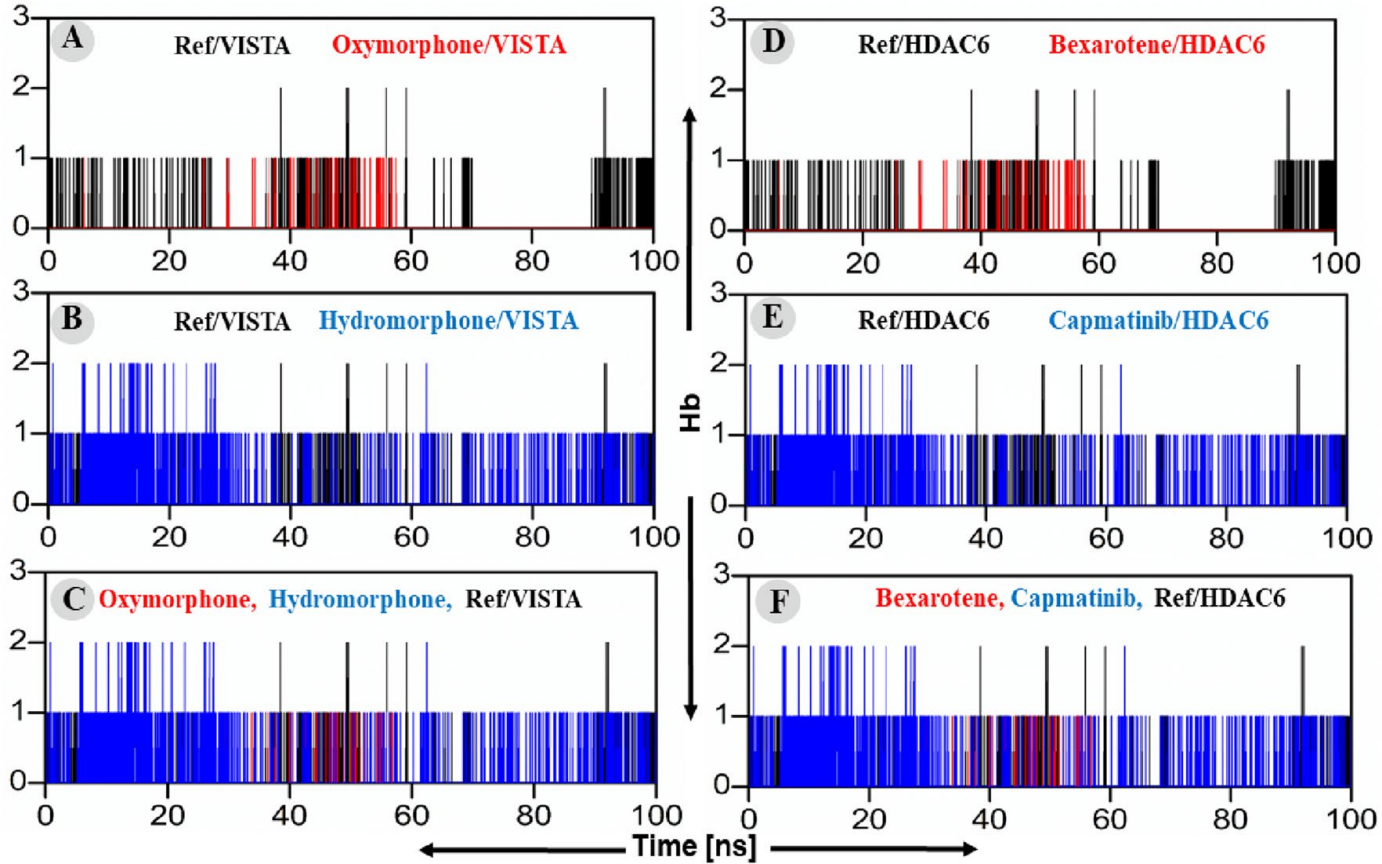
(**A**–**C**) Plot of the hits/VISTA and ref-drug (CA-170)/VISTA complexes’ hydrogen bonding. The x-axis and y-axis showed the time in nanoseconds (100 ns) and hydrogen bonding in Angstroms respectively. (**A**) Hydrogen bonding of Oxymorphone–VISTA complex (red) and CA-170/VISTA complex (black), (**B**) Hydromorphone–VISTA complex (Blue) and CA-170/VISTA complex (Black) Hydrogen bonding, (**C**) Combine Hydrogen bonding compassion of VISTA. While (**D**–**F**) plot of the hits/HDAC6 and ref-drug (Bexarotene)/HDAC6 complexes’ root mean square deviations. (**D**) Hydrogen bonding of Bexarotene–HDAC6 complex (red) and Trichostatin A/HDAC6 (black), (**E**) Hydrogen bonding of Capmatinib–HDAC6 complex (Blue) and Trichostatin A/HDAC6 (black), (**F**) Combine Hydrogen bonding compassion of HDAC6.

Hydrogen bond analyses is an important parameter to understand the binding affinity and stability of protein–ligand complex in drug discovery platform^37^. Herein, the simulation results of our study showed that Oxymorphone retained a single hydrogen bond with the binding pocket residues VISTA at the middle of simulation over a period of almost 20 ns as shown in Fig. 6A, left panel. On the other hand, the Hydromorphone retained a single hydrogen bond with VISTA during the entire simulation period (Fig. 6B, left panel). The superimposed view of the hydrogen bond pattern of the reference compound, Oxymorphone and Hydromorphone with the binding pocket residues of VISTA are shown in Fig. 6C, left panel. In parallel, the simulation results of Bexarotene in complex with HDAC6 showed that Bexarotene could retain a single hydrogen bond with the binding pocket residues of HDAC6 over a short span of simulation (Fig. 6D, right panel). In contrast, the Capmatinib established and retained a single hydrogen bond with HDAC6 over the entire simulation period as shown in Fig. 6E, right panel. The superimposed view of the hydrogen bond pattern of the reference compounds, Bexarotene and Capmatinib with the binding pocket residues of HDAC6 are displayed in Fig. 6F, right panel.

#### Principal components analyses and free energy landscape analysis (PCA and FEL)

**Figure 7:**
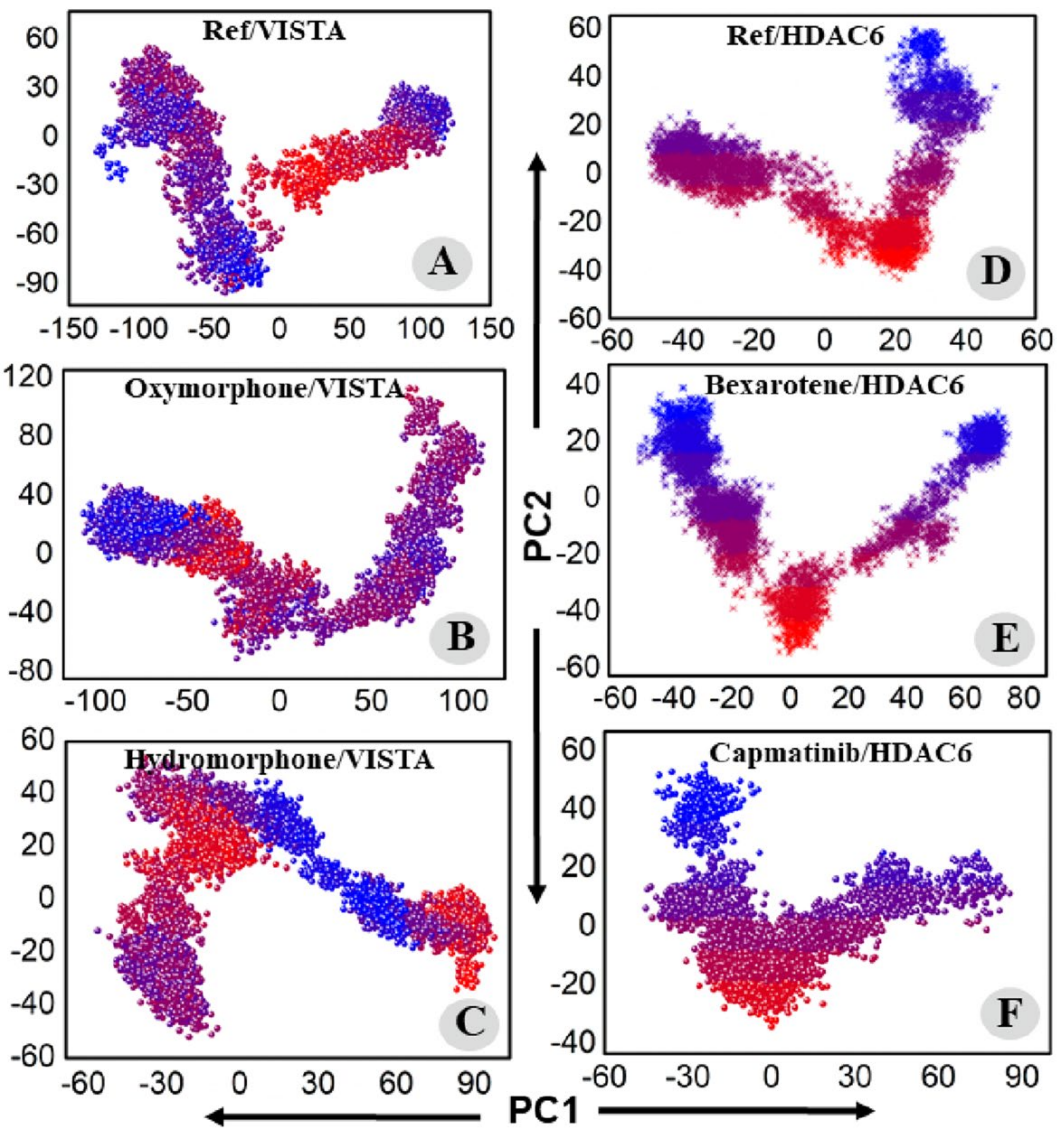
(**A**–**C**) PCA plots of the Ref/VISTA, Oxymorphone–VISTA, Hydromorphone–VISTA While (**D**,**E**) plot of the ref-drug/HDAC6, Bexarotene–HDAC6, and Capmatinib–HDAC6 complex.

**Figure 8:**
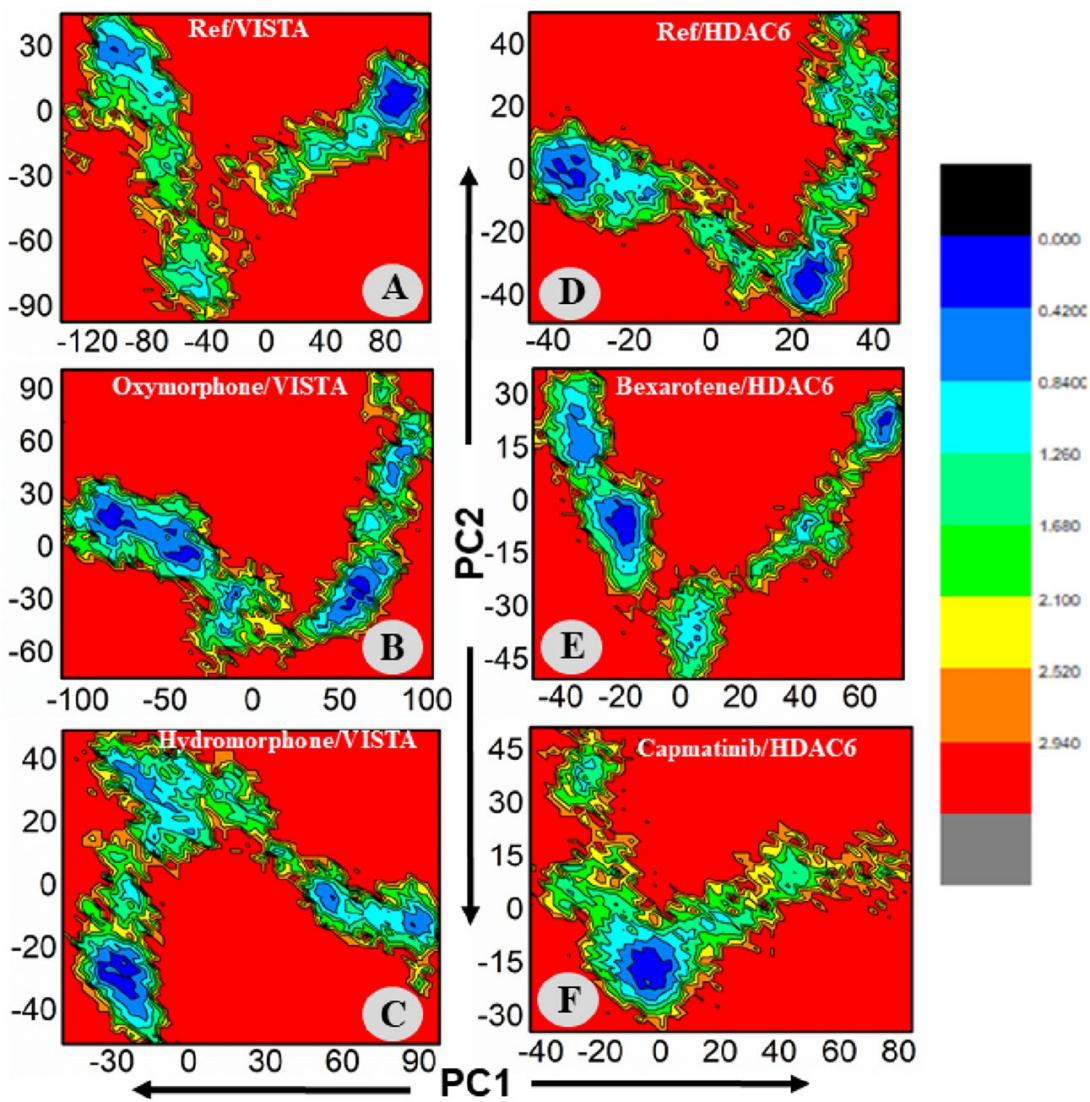
(**A**–**C**) FEL plots of the Ref/VISTA, Oxymorphone–VISTA, Hydromorphone–VISTA. While (**D**,**E**) plot of the ref-drug/HDAC6, Bexarotene–HDAC6, and Capmatinib–HDAC6 complex.

To capture the most dominant motions during MD simulation PCA was applied to the coordinate covariance matrix derived from 100 ns MD simulation of lead compound complexes. The first two principal components (PC1 and PC2) which captures most of the motions were plotted. Here, we performed the principal VISTA and ref-drug (CA-170), Hydromorphone–VISTA complex, Oxymorphone–VISTA complex, ref-drug (Trichostatin A)/HDAC6, Bexarotene–HDAC6 complex, Trichostatin A/HDAC6, Capmatinib–HDAC6 complex, and Trichostatin A/HDAC6 complexes. The color gradient (blue to red) in the figure highlights the periodic changes in conformation. According to PCA each complex had different motion patterns. The ref compound's motion was slightly dispersed (Fig. 7A), with blue dots at the start, and red at the end. In contrast, compound Oxymorphone display cluster and compact type of motion compared to the ref (Fig. 7B), covering a range of − 100 to + 100 along PC1 and − 80 to + 120 along PC2. The second inhibitor Bexarotene also revealed cluster type of motion (Fig. 7C), covering a range of − 60 to + 80 at PC1 and − 60 to + 340 at PC2. The PCA analysis of all the complexes are displayed in Fig. 7. Further, the first two eigenvectors were used to calculate the free energy landscape (FEL) of VISTA and HDAC6 in complex with bound drugs. In order to comprehend the structural dynamics of each complex, the dominant and metastable state conformations were calculated from the FEL during the 100-ns MD simulation (Fig. 8). The lowest Gibbs energy states are indicated by the blue color. Different colors were used in the plot to depict high and low-energy states. Blue color indicates a low energy level, while red color indicates a high energy state. The blue color is more prevalent in ligands Oxymorphone–VISTA complex, Hydromorphone–VISTA, Bexarotene–HDAC6, and Capmatinib–HDAC6 complex, which demonstrate high stability of these drugs during MD simulation.

### Binding free energy calculation

The binding free energy (ΔGbind) is calculated as the difference in the solvation free energy of the complex and the solvation free energy of the individual components. The non-bonded energy (ΔGnon-bonded) is calculated as the difference in the non-bonded interactions between the complex and the individual components. Molecular Mechanics Generalized Born Surface Area (MMGBSA), a well-known technique, has been used to accurately predict the binding free energies of all the complexes. Gibbs free energy controls the binding affinity between the interacting molecules^38,39^. The total binding free energies of all the retrieved hits along with the reference complex as well as van der Waals, electrostatic energy, polar solvation energy and other energies terms. These finding strongly support that our retrieved hits had greater potential for inhibition than the reference compounds. The active lead in the database Bexarotene/HDAC6, had an even lower MMGBSA score of − 51.9698 ± 3.1572 kcal/mol, Capmatinib/HDAC6 showed − 48.8880 ± 5.7236, and ref/HDAC6 showed − 18.0253 ± 2.6218 while Oxymorphone/VISTA, Hydromorphone/VISTA, and Ref/VISTA showed the MMGBSA score − 36.8323 ± 3.4565, − 25.7331 ± 1.1980, and − 21.5611 ± 4.8581 respectively. Based on these results, it can be deduced that the hits that were retrieved had good free binding energy score (average ± standard error of the mean (SEM)) than the reference compound.

## Discussion

Cancer immunotherapy is paying much attention in the recent years with the accumulating evidences elucidating the exceptional role of immune system to defend body from cancer initiation, progression and metastasis^40^. When cancer cells invade an intact tissue, the immune system if recognized such transformed cells as cancerous should eradicate cancerous cells^41,42^. Immune system does so by reactivating the inhibited immune cells weapons to generate sufficient anti-cancer actions to eradicate the invading cancerous cells. Cancer immunotherapy empowers the braked cytokines, chemokines as well as certain immune cells to reshape the tumor microenvironment limiting the aggressive behavior of cancer^43^. It is well known that deep epigenetic changes occur within cancer cells that aids in its progression. One of the affected enzymes are HDAC6, a novel histone deacetylase that is present in the cytoplasm hydrolyzing non-histone proteins^44^. It is deemed a significant contributor to the excessive proliferation of cancerous cells and, thus, was the targets of many trials. Its inhibition displayed relative successful that benefited even standard treatment-resistant types of cancers^45^. Similarly, traditional ICI like PD-L1 and CLTA-4 was also resisted by cancerous cells. On the flip side, VISTA, a negative immune regulator, is unique in its expression in naïve cells giving more opportunity of the newly formed immune cells to eradicate cancer tissue^46^. Additionally, only few trials are considering VISTA as a potential ICI target^47^. Therefore, the aim of this study is to screen for potential repurposable FDA-approved drugs against HDAC6 (braking the cancer progression) and VISTA (removing the imposed immunosuppression) as a novel dual therapeutic strategy for cancer treatment.

The results of the present study found that many FDA-approved drugs can replace the reference inhibitor of HDAC6 as they displayed stronger binding affinity. The ADME features of the top-10 drugs satisfy Lipinski's rule of five appropriately. The detailed enzyme-drug interaction unveiled the fit to the active site with the formation of numerous H-bonds accounting for the stronger binding affinity. The best HDAC6 potential drug was Bexarotene. Bexarotene is already in use for prevention, treatment as well as alleviating the drug-resistance of conventional chemotherapy^48^. However, its use is restricted to cutaneous T-cell lymphoma and in the phase I of breast cancer clinical trial^49^. On the other side, the best repurposable drug prediction to inhibit VISTA was Oxymorphone (binding affinity of − 6.2 kcal/mol) with excellent pharmacokinetics profile. Oxymorphone is a typical mu-opioid agonist that is effective in both immediate- and extended-release formulations designed for pain relief^50^. Therefore, coupling the blocking of cancer epigenetic regulator (HDAC6) and ICI (VISTA) with Bexarotene and Oxymorphone is an unprecedented way to aggressively limit cancer pathogenicity that deserves experimental validation.

## Conclusion

In the present study virtual screening of FDA approved drugs retrieved from the DrugBank database was carried out against the HDAC6 and VISTA cancer drug targets. Different computational tools were used in this study which leads to the identification of drugs against cancer. In terms of docking score, stability, and MMGBSA calculation the two drugs Bexarotene and Oxymorphone were found as the most effective drugs among all the drugs of the DrugBank database. Overall, the two drugs Bexarotene and Oxymorphone revealed the strong binding affinity toward the cancer drug targets HDAC6 and VISTA. These drugs can be helpful to treat cancer. However, to validate the results of our investigation, additional in vitro and in vivo studies are recommended.

## Data Availability

The data presented in this study are available on request from the corresponding author.
